# Clinical Assessment on Days 1–14 for the Characterization of Traumatic Brain Injury: Recommendations from the 2024 NINDS Traumatic Brain Injury Classification and Nomenclature Initiative Clinical/Symptoms Working Group

**DOI:** 10.1089/neu.2024.0577

**Published:** 2025-07-09

**Authors:** David K. Menon, Noah D. Silverberg, Adam R. Ferguson, Thomas J. Bayuk, Shubhayu Bhattacharyay, David L. Brody, Scott A. Cota, Ari Ercole, Anthony Figaji, Guoyi Gao, Christopher C. Giza, Fiona Lecky, Rebekah Mannix, Ana Mikolić, Kasey E. Moritz, Claudia S. Robertson, Abel Torres-Espin, Spyridoula Tsetsou, John K. Yue, Hibah O. Awad, Kristen Dams-O’Connor, Adele Doperalski, Andrew I.R. Maas, Michael A. McCrea, Nsini Umoh, Geoffrey T. Manley

**Affiliations:** ^1^ Department of Medicine, University of Cambridge, Addenbrooke’s Hospital, Cambridge, UK.; ^2^ Department of Psychology, University of British Columbia, Vancouver, British Columbia, Canada.; ^3^ Rehabilitation Research Program, Centre for Aging SMART, Vancouver Coastal Health Research Institute, Vancouver, British Columbia, Canada.; ^4^ Djavad Mowafaghian Centre for Brain Health, University of British Columbia, Vancouver, British Columbia, Canada.; ^5^ Brain and Spinal Injury Center (BASIC), Department of Neurological Surgery, University of California San Francisco, San Francisco, California, USA.; ^6^ San Francisco Veterans Affairs Healthcare System, San Francisco, California, USA.; ^7^ Department of Neurology, Uniformed Services University of the Health Sciences, Bethesda, Maryland, USA.; ^8^ Harvard Medical School, Boston, Massachusetts, USA.; ^9^ Uniformed Services University of the Health Sciences, Bethesda, Maryland, USA.; ^10^ Former Branch Chief Traumatic Brain Injury Center of Excellence (TBICoE DHA), Biloxi, Mississippi, USA.; ^11^ Division of Anaesthesia, University of Cambridge. Cambridge, UK.; ^12^ Paediatric Neurosurgery, Red Cross War Memorial Children’s, Hospital Neurosciences Institute, University of Cape Town, Cape Town, South Africa.; ^13^ Department of Neurosurgery, Beijing Tiantan Hospital, Capital Medical University, Beijing, China.; ^14^ Departments of Pediatrics and Neurosurgery, UCLA Brain Injury Research Center, UCLA Mattel Children’s Hospital, Los Angeles, California, USA.; ^15^ David Geffen School of Medicine at UCLA, Los Angeles, California, USA.; ^16^ School of Medicine and Population Health, University of Sheffield, Shefield, UK.; ^17^ Division of Emergency Medicine, Boston Children’s Hospital, Harvard Medical School, Boston, Massachusetts, USA.; ^18^ Department of Psychology, University of British Columbia, Vancouver, British Columbia, Canada.; ^19^ Combat Casualty Care Research Program, US Army Medical Research and Development Command, Fort Detrick, Maryland, USA.; ^20^ Department of Neurosurgery, Baylor College of Medicine, Houston, Texas, USA.; ^21^ School of Public Health Sciences, University of Waterloo, Waterloo, Ontario, Canada.; ^22^ Department of Neurological Surgery, Brain and Spinal Injury Center, University of California San Francisco, San Francisco, California, USA.; ^23^ Department of Neurology and Neurosurgery, Baylor College of Medicine, Houston, Texas, USA.; ^24^ Weill Institute for Neurosciences, School of Medicine, University of California San Francisco, San Francisco, California, USA.; ^25^ Division of Neuroscience, National Institute of Neurological Disorders and Stroke, Bethesda, Maryland, USA.; ^26^ Department of Rehabilitation and Human Performance, Icahn School of Medicine, New York, New York, USA.; ^27^ Department of Neurology, Icahn School of Medicine, Mount Sinai, New York, New York, USA.; ^28^ Division of Neuroscience, National Institute of Neurological Disorders and Stroke, Bethesda, Maryland, USA.; ^29^ Department of Neurosurgery, Antwerp University Hospital, Edegem, Belgium.; ^30^ Department of Translational Neuroscience, Faculty of Medicine and Health Science, University of Antwerp, Antwerp, Belgium.; ^31^ Department of Neurosurgery, Medical College of Wisconsin, Milwaukee, Wisconsin, USA.; ^32^ Division of Neuroscience, National Institute of Neurological Disorders and Stroke, Bethesda, Maryland, USA.; ^33^ Neurological Surgery, University of California San Francisco, San Francisco, California, USA.

**Keywords:** CBI-M framework, classification and characterization, craniocerebral trauma, diagnostic techniques and procedures, Glasgow Coma Scale, NIH NINDS, pupil disorders, prognosis, traumatic brain injury

## Abstract

The current classification of traumatic brain injury (TBI) primarily uses the Glasgow Coma Scale (GCS) to categorize injuries as mild (GCS 13–15), moderate (GCS 9–12), or severe (GCS ≤8). However, this system is unsatisfactory, as it overlooks variations in injury severity, clinical needs, and prognosis. A recent report by the National Academies of Sciences, Engineering, and Medicine (USA) recommended updating the classification system, leading to a workshop in 2024 by the National Institute of Neurological Disorders and Stroke. This resulted in the development of a new clinical, biomarker, imaging, and modifier (CBI-M) framework, with input from six working groups, including the Clinical/Symptoms Working Group (CSWG). The CSWG included both clinical and non-clinical experts and was informed by individuals with lived experience of TBI and public consultation. The CSWG primarily focused on acute clinical assessment of TBI in hospital settings, with discussion and recommendations based on pragmatic expert reviews of literature. Key areas reviewed included: assessment of neurological status; performance-based assessment tools; age and frailty, pre-existing comorbidities, and prior medication; extracranial injuries; neuroworsening; early physiological insults; and physiological monitoring in critical care. This article reports their discussions and recommendations. The CSWG concluded that the GCS remains central to TBI characterization but must include detailed scoring of eye, verbal, and motor components, with identification of confounding factors and clear documentation of non-assessable components. Pupillary reactivity should be documented in all patients, but recorded separately from the GCS, rather than as an integrated GCS-Pupils score. At ceiling scores on the GCS (14/15), history of loss of consciousness (LoC) and the presence and duration of post-traumatic amnesia should be recorded using validated tools, and acute symptoms documented in patients with a GCS verbal score of 4/5 using standardized rating scales. Additional variables to consider for a more complete characterization of TBI include injury mechanism, acute physiological insults and seizures; and biopsychosocial-environmental factors (comorbidities, age, frailty, socioeconomic status, education, and employment). The CSWG recommended that, for a complete characterization of TBI, disease progression/resolution should be monitored over 14 days. While there was a good basis for the recommendations listed above, evidence for the use of other variables is still emerging. These include: detailed documentation of neurological deficits, vestibulo-oculomotor dysfunction, cognition, mental health symptoms, and (for hospitalized patients) data-driven integrated measures of physiological status and therapy intensity. These recommendations are based on expert consensus due to limited high-quality evidence. Further research is needed to validate and refine these guidelines, ensuring they can be effectively integrated into the CBI-M framework and clinical practice.

## Introduction

This report from the Clinical/Symptoms Working Group (CSWG) of the 2024 National Institute of Neurological Disorders and Stroke (NINDS) TBI Classification and Nomenclature Workshop (hereafter referred to as the “Workshop”) provides a narrative expert review and pragmatic recommendations for the acute clinical characterization of patients with traumatic brain injury (TBI). We describe composition, processes, and findings of the Clinical/Symptoms Working Group. An overview of the new framework for the characterization of TBI and the NINDS TBI classification and nomenclature initiative is provided elsewhere.^1^ In brief, the initiative is responsive to the National Academies of Sciences, Engineering, and Medicine (NASEM)’s 2022 consensus study report titled *Traumatic Brain Injury: A Roadmap for Accelerating Progress* (https://nap.nationalacademies.org/login.php?record_id=25394), which listed as its first recommendation: “Create and implement an updated classification system for TBI.” One of the aims of the initiative is to move from the conventional TBI severity classification of mild/moderate/severe in the acute phase based on a single assessment metric, to a richer classification characterized based on four pillars: clinical findings, biomarkers, imaging, and modifiers (a clinical, biomarker, imaging, and modifier [CBI-M] scheme). The present report from the CSWG should be viewed alongside those from the other five working groups involved in this initiative, and published in this special issue of the *Journal of Neurotrauma*. These include articles from working groups addressing: neuroimaging^2^; blood-based biomarkers^3^; psychosocial and environmental modifiers^4^; knowledge to practice^5^; and retrospective classification.^6^

The Glasgow Coma Scale (GCS)^7^ has been most widely used to clinically assess TBI severity. It was developed in 1974 as a standardized tool for assessing the level of consciousness after TBI but evolved into the gold-standard method for documenting and communicating overall TBI severity.^8^ Despite its well-known limitations for this purpose,^9^ it is enduring into its sixth decade. Traditionally, the total score is trichotomized into mild (GCS = 13–15), moderate (GCS = 9–12), or severe (GCS ≤8) classification.^10–12^ CSWG concluded that the GCS remains a very useful tool for TBI characterization; however, there is an opportunity to use it more effectively (e.g., report subscores and avoid trichotiomization into mild/moderate/severe categories). The CSWG further concluded that supplemental clinical assessments are important, especially to characterize patients with GCS floor and ceiling scores.

The CSWG recognized that a wide range of assessment instruments and clinical findings other than the GCS have been used to document TBI severity. Two principles guided CSWG decision about which information to include and prioritize for implementation. First, the CSWG prioritized assessments that inform clinical decision-making in the acute care hospital setting, that is, those that drive triage, establish a diagnosis of TBI, optimize resuscitation targets to minimize secondary injury, trigger diagnostic interventions (such as blood biomarkers and neuroimaging), allocate patients to clinical pathways (discharge from the emergency department [ED], or admission to a hospital ward or intensive care unit [ICU]), and determine follow-up needs. Second, the CSWG prioritized assessments with prognostic import, harking back to the original goal of TBI severity classification.

The primary focus of the CSWG was clinical characterization of the severity of TBI, after TBI has been clinically diagnosed or suspected. There is an overlap between clinical assessment findings that indicate the presence of TBI and those that help grade its severity. For example, LoC immediately following head trauma is a sign that can rule-in a TBI diagnosis,^13–15^ while its presence and duration contribute to defining TBI severity. Although diagnosing TBI and then characterizing its severity is a logical sequence, following that order is not always possible. Diagnostic challenges often arise when it is difficult to confirm whether LoC and/or amnesia had actually occurred at the time of injury (classically in the following cohorts: patients with pre-injury cognitive impairment, young children), whether any amnesia that is documented is not conclusively attributable to TBI (such as in patients with pre-injury intoxication), and/or instances where a comprehensive assessment is impossible because of therapeutic interventions (such as sedation and tracheal intubation). In such cases, TBI may be “suspected”^15^ while injury characterization proceeds, such as with blood-based biomarkers and neuroimaging.

## Methods

### Working Group membership

CSWG consisted of 22 individuals (Supplementary Data) from five countries, including clinicians with expertise in adult, older adult, and pediatric populations, non-clinical researchers, and representatives of U.S. federal government agencies. The CSWG included members with a wide range of clinical and non-clinical expertise covering emergency medicine, neurosurgery, critical care, neurology, neuropsychology, and epidemiology. Our work was substantially informed and influenced by individuals with lived experience of TBI, public feedback, and discussions with other working groups before, during, and after the Workshop. In particular, their input highlighted the need for a more complete and nuanced characterization of TBI, rather than a crude classification as mild, moderate, and severe. We heard powerful testimony from these individuals about how such a crude classification could lead to “mild TBI” being dismissed as trivial (despite the common incidence of incomplete recovery at 6 months), while “severe TBI” might engender therapeutic nihilism and lose opportunities for good outcomes with aggressive management.

Of the 22 individuals in the CSWG, 18 substantive members were invited to participate, based on experience and expertise, through discussions with the Workshop Steering Committee (two of these were from low/middle-income countries, and three had specific experience in pediatric TBI). The remaining four members were early career researchers, who were initially inducted to the CSWG as Associate Members, but contributed substantially to the process and are recognized here as full authors of this article.

### Scope

The scope of the work described in this article was decided by CSWG Chairs (D.K.M., N.D.S., A.R.F.) in discussion with the Workshop Steering Committee, and undertaken in the wider context of the NINDS TBI Classification and Nomenclature Workshop. We recognized that a large proportion of patients who sustain a TBI never present to the hospital. However, given the framework of the workshop, and given considerations of practicality, we agreed that the scope for our work would be limited to clinical assessment of TBI in patients presenting to the hospital.

Conventionally, the categorization of severity (and hence patient care and prognostic impressions) of TBI in the hospital setting has depended on initial assessment at presentation. However, TBI evolves over minutes, hours, days,^16^ and weeks.^17–19^ Therefore, it is not possible to fully assess the effects of TBI at a single early timepoint (e.g., ED arrival),^20^ and patients who initially look similar (e.g., GCS = 13 with subdural hematoma) may follow diverging trajectories after initial triaging assessment. Exploration of the full disease narrative of TBI was beyond the scope of our work. However, given this background, the CSWG aimed to cover assessment both at the point of hospital presentation (typically to an ED) and any evolution of clinical features over the first 14 days post-TBI, which informed diagnosis, triage, investigations, clinical interventions, and prognosis across the severity range in TBI. Trauma (and by extension TBI) is the leading cause of death for children in the United States (and many other parts of the world), but a detailed discussion of pediatric TBI was precluded by practical considerations regarding collation and analysis of further tranche of literature and article length. Consequently, while this article makes reference to pediatric TBI in several areas, the primary focus of this article is on adult TBI, and where appropriate, highlights issues pertaining to TBI in older adults.

### Process

The CSWG met online on eight occasions (between June 2023 and January 2024) preceding the Workshop held at Bethesda, MD, on the 22nd and 23rd of January 2024. The work of the Working Group was informed by expert pragmatic reviews of the literature, led by one to three of the Working Group members with specific expertise on the topic, followed by detailed discussions of their evidence summaries in the Working Group meetings. Reviews covered the following topics:

• Clinical assessment of neurological status
• Performance-based assessment tools at the mild end of the severity spectrum
• Assessing and quantifying the impact of frailty, pre-existing comorbidity, and medication
• Characterizing extracranial injury and its impact on clinical care and prognosis
• Neuroworsening and discharge decisions for hospitalized patients
• Physiological monitoring in critical care
• Detection and quantification of physiological insults
• In addition, the CSWG prepared briefing notes outside this scope of work in specific areas where an evidence summary was thought to be useful and members or the CSWG had specific expertise, but these are not included in the current article. These were:
  o Clinical assessment of patients with TBI who do not present to the hospital; and
  o New approaches to data acquisition, management, and analysis

The outputs of this process were presented at (and informed by discussions at) Workshop Steering Committee meetings (which included other Working Group Chairs) and a draft Summary Document was generated. This summary document was informed by discussions with members of the Knowledge to Practice Working Group, members of which attended one of our later Working Group meetings. Overlaps with other working groups (e.g., the “Modifiers” Working Group) were addressed through presentations of our plans at their meetings. The resulting modified Summary Document was circulated to the full membership of the other Working Group, and following feedback, a final Summary Document was placed online for public consultation on the Workshop Sharepoint. The recommendations in this document were presented at the Workshop in Bethesda, and informed by general discussion, and by the powerful personal testimony of the panel of individuals with lived experience of TBI. The Summary Document was further modified by a post-Workshop discussion of the CSWG in Bethesda, public feedback, subsequent online discussions of the Working Group, and discussions with the Workshop Steering Committee. These discussions led to a more detailed analysis of two areas—the possible integration of pupillary reactivity and GCS into a single score, and the use of single assessments of post-traumatic amnesia (PTA) as part of the acute assessment of TBI. These analyses were undertaken in large observational datasets and have been published elsewhere, but the results are summarized in our article. The current article is informed by this series of post-Workshop discussions.

## Findings

### Context

**FIG. 1.**
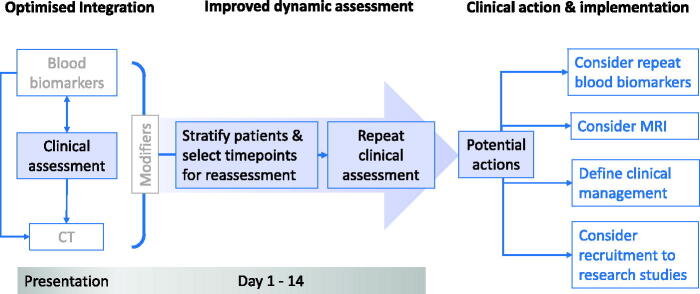
Clinical assessment as part of clinical/biomarker/imaging-modifiers (CBI-M) assessment framework. Conceptually, initial clinical assessment would (in most cases) form the first step in a comprehensive characterization and inform need for repeat blood-based biomarker measurement and neuroimaging (including MRI), allocation to clinical care pathways, arrangements for follow-up, and prognostic expectations. This initial assessment would be enriched by a dynamic assessment of clinical progress over the first 2 weeks following TBI. Integration of information from such assessment would inform not just investigations and clinical care but also potential recruitment to research studies. We recognize that while other biofluids such as saliva and sweat may provide options for biomarker analysis in the future, we have primarily addressed the use of blood biomarkers in this scheme as those are closest to routine clinical practice. MRI, magnetic resonance imaging; TBI, traumatic brain injury.

The CSWG recommends that any clinical assessment should be part of a comprehensive scheme that informs and is part of the characterization of TBI (Fig. 1). The clinical examination (C), along with blood biomarkers (B), and neuro-imaging (I) are three key pillars for description of TBI, and along with a consideration of modifying factors (M), provided an integrated CBI-M framework for characterizing TBI. The process undertaken by the CSWG resulted in a refinement of the focus of our discussion of the available evidence under the following headings: objective clinical examination of neurological status; mechanisms of injury; TBI symptoms; objective assessment of cognition, balance, and vestibulo-oculomotor dysfunction; extracranial injury; early physiological insults; age, comorbidities, and frailty; concurrent therapy; and dynamic assessments. Each of these is discussed separately below.

#### Objective clinical examination of neurological status

##### The Glasgow Coma Scale Score

The GCS is a highly pragmatic tool for documenting whether a patient has overtly normal alertness and clarity of thinking (GCS = 15), or presents with confusion (GCS = 14; GCS-V = 4, a core feature of “altered mental status”^15^), or reduced consciousness, including coma (GCS <9). However, the current use of the GCS, particularly in trichotomization of the total score (to “mild,” “moderate,” and “severe” TBI) has flaws. First, there is considerable variability in injury severity, treatment needs, and prognosis within each of the crude mild (13–15), moderate (9–12), and severe (≤8) categories. Information is better communicated by stating the actual GCS, ideally specifying subcomponent contributions, since these have different clinical and prognostic relevance. Second, the GCS has significant floor/ceiling properties, and imprecision is worst at extremes (sum scores of 3 and 15).^21^ Third, the application of the GCS requires modification in preverbal children^22^ and can be affected by pre-injury cognitive deficits.^23^

It is critical to ensure that the procedures used to score the GCS follow standard approaches^7^ (see also https://www.glasgowcomascale.org/ for details and instruction videos). Careful and consistent attention to detail is essential—for example, when the motor component is being scored, this is typically done in the upper extremities, with the response of the better arm recorded as the motor response.

Such conventional clinical assessment works to identify the need for CT brain imaging, early neurosurgery or critical care interventions, and appropriate post-ED clinical pathway selection. This approach reliably detects neurosurgical lesions or pathology requiring other immediate attention.^24^ However, most patients present with normal or minimally impaired consciousness and a “normal CT.” There is a common impression that a normal CT makes “clinically significant” TBI highly unlikely, and specific follow-up and rehabilitation is unnecessary. We now know that this view is unduly optimistic: many of this “mild” group who present to Level 1 Trauma Centers and meet thresholds for CT imaging suffer ongoing disabling symptoms.^25^ Conversely, patients who present with a lower GCS may be subject to therapeutic nihilism and premature withdrawal of life-sustaining therapy (WLST),^26,27^ despite the fact that GCS assessment at presentation (particularly in patients with a sum GCS of 3) may be falsely lowered by alcohol, recreational drugs, sedative medication or tracheal intubation; or confounded by a post-ictal state or systemic physiological derangements (issues discussed in more detail in subsequent sections of this article). Further, a propensity-matched analysis suggests that a third of these patients might, with continued aggressive therapy, survive to independent recovery.^28^ Additional assessment tools are needed to address these confounds.

##### Pupillary reactivity

Pupillary reactions to light provide a strong clinical biomarker that informs patient management and prognostication,^29^ suggesting brainstem compression due to a space-occupying lesion or brain swelling, and while peripheral ocular or cranial nerve injury must be excluded, they have strong prognostic import. It can be difficult to robustly diagnose the lack of pupillary reactivity to light by simple observation, especially when pupils are small, and automated pupillometry may provide a more consistent assessment.^30^

It has been suggested^31^ that subtracting a point from the GCS for each unreactive pupil could generate an integrated “GCS-Pupils score (GCS-P)”. This provides a characterization of patients below a conventional GCS floor of 3, where patients lose an additional point for each unreactive pupil. Thus, patients with a conventional GCS score of 3 and one unreactive pupil would be scored as GCS-P = 2, and those with both unreactive pupils scored as GCS-P = 1. While conceptually appealing, there are both statistical disadvantages and practical difficulties with this approach:^32^

• The prognostic information provided by the GCS-P score is greater than the sum GCS on its own, but this increment is only about half of that provided by including the pupillary responses as separate variables (Supplementary Fig. S1)
• At a GCS ≥3, a summary GCS-P score is necessarily ambiguous about whether points are lost for pupils or another GCS components (Supplementary Fig. S2)– and this ambiguity affects both likely prognosis and clinical decision-making.

This discussion leads us to strongly recommend assessing and recording pupillary responses to light, using automated pupillometry if possible, but record these separately from the GCS, rather than as a combined GCS-P score.

##### Timing and confounding factors

These clinical assessments may vary significantly in the immediate post-injury period (either improvement after the initial ictus or clinical deterioration (“neuroworsening”; see later), which makes them subject to substantial variability depending on when they are measured. Furthermore, early data from the pre-hospital setting may rely on first responders, in which context assessment levels of consciousness may be less reliable and reproducible. Thus, reliable data are often missing early in the patient’s course. The best initial GCS might reasonably be expected to most faithfully capture the severity of the initial injury. However, it may also be confounded by hypoxia or hypotension, but the concept of “best resuscitated” GCS is not relevant to modern pre-hospital practice where resuscitation and stabilization may all occur concurrently and frequently require sedation and endotracheal intubation. Therefore, while later assessments of GCS by more experienced practitioners might be expected to be more complete, for the more severely injured patients, these might not be fully assessable as these patients would likely be intubated, sedated, paralyzed, and mechanically ventilated.

We are, therefore, faced with how to best use GCS/pupillary responses assessed at a variety of timepoints, many of which might be missing. Imputation strategies have been examined using data from the CENTER-TBI dataset.^33^ Where we need to choose a “most predictive” neurological assessment from variably missing data, a substitution strategy is needed but there are a variety of possible choices as to which GCS/pupillary assessment to choose. Model performance (in terms of pseudo-R2 explained “pseudovariance”) varies somewhat with both substitution strategy and type of model (e.g., dichotomous vs. ordinal regression) and so the choice is not an entirely trivial one.

One approach to the confound of tracheal intubation has been to limit assessment to the motor subscore (which is strongly prognostic in patients with GCS 3–12).^34^ However, in the overall TBI population, the GCS sum performs better than the motor score alone overall, so there is a case for exploring alternative strategies.^35^ Of a variety of choices, the strategy used by the International Mission for Prognosis and Analysis of Clinical Trials in TBI (IMPACT)^34^ generally works well and is simple: the ED discharge assessment GCS/pupil assessment is used. If this is missing, this is substituted with an alternative available value, moving backwards in the patient’s clinical course (i.e., ED discharge → study hospital ED arrival → referring hospital ED arrival → pre-hospital).^33^

One uncertainty is that pupillary responses may remain assessable even when the GCS is not, and this could lead to discordances in the time of recording. For example, a patient with an expanding extradural hematoma may have had a well-documented GCS of 15 at the scene of the injury, but subsequently show neuroworsening, develop an unreactive pupil, and require sedation and tracheal intubation. In this context, the GCS is un-assessable due to sedation and a sedation hold is inappropriate. In these circumstances, it would seem inappropriate to combine the most robustly documented GCS (of 15) with report of an unreactive pupil. The CSWG concluded that it might be best to record the last GCS before intubation, which integrates the effect of neuroworsening.

Where a GCS component is untestable, several options are available, but the amount of detail that can be obtained is always constrained by the burden of data recording. In the past, un-assessed motor and verbal scores hc as 1—but this is not optimal.^33^ An alternative,^7^ which we would recommend, is not to score it as 1, but rather to specify this, and amend notation to this effect. For example, in the past, the notation of “V(t)” has been used in an intubated patient. However, there may be other constraints to full GCS assessment, and if a complex notation system is to be avoided, perhaps all untestable components could be specified with a single notation (“U”: e.g., V(U)), which in subsequent group level research analyses could mark scores for imputation. However, such usage (on its own) makes calculation of a sum GCS impossible (since there is no score attached to an untestable component). A compromise would be to score the untestable component as “1” for calculation of a sum score, and append the “U” as a suffix (e.g., GCS 3U, broken down as E(U) V(U) M(U) for a sedated and intubated patient).

Less binary confounds (e.g., sedation, a recent seizure) may allow some scoring, and it is not clear what the best approach should be. In research settings, well-justified imputation may be the best option. However, it remains unclear which of these strategies is optimal, and the best options may vary depending on whether the goal is individual TBI characterization in clinical practice, or analysis of a research dataset.

##### Alternatives to the GCS

Alternative severity classification and diagnostic schemes (e.g., FOUR Score,^36^) are not optimal approaches for characterizing TBI across the severity spectrum in the acute phase. Similarly, while the presence or absence of LoC is often recorded, there is inconsistent evidence that this binary variable provides prognostic information, so the CSWG would not recommend it in isolation.^37,38^ The Alert, responds to Voice, responds to Pain, Unresponsive (AVPU) scale is commonly used in pre-hospital and emergency care and provides a more nuanced, but still unitary measure of consciousness. While the AVPU score does correlate with the GCS, very few studies have examined its use in early TBI assessment.^39^

##### Detailed neurological examination

Detailed evaluation of neurological function (e.g., lateralizing weakness, language deficits, ocular dysfunction, cerebellar signs) is recommended as part of a comprehensive clinical examination,^40^ and ongoing neurological monitoring can inform diagnosis and management decisions (see “*neuroworsening*,” later). However, these assessments are not systematically and consistently performed or documented in the acute care setting in the ED. It is very likely that at least some of these assessments are important in the early characterization of TBI, but the systematic incorporation of these assessments into clinical care, and recommending the best format for recording their findings, will require further evaluation of their sensitivity, specificity, reliability, and robustness in the ED setting.

### Post-traumatic amnesia

The duration of post-traumatic amnesia (PTA), especially in the range of 1–60 days, is highly prognostic when assessed prospectively with validated tools,^41^ such as the Galveston Orientation and Amnesia Test (GOAT), Westmead PTA Scale, or O-Log. A study directly comparing these three instruments found that the Westmead PTA Scale took the longest to normalize after TBI, whereas patients who passed the other PTA tests often had ongoing agitation and memory impairment, suggesting persisting PTA.^42^ The international INCOG expert group recommended the Westmead scale for PTA assessment.^41^

**FIG. 2.**
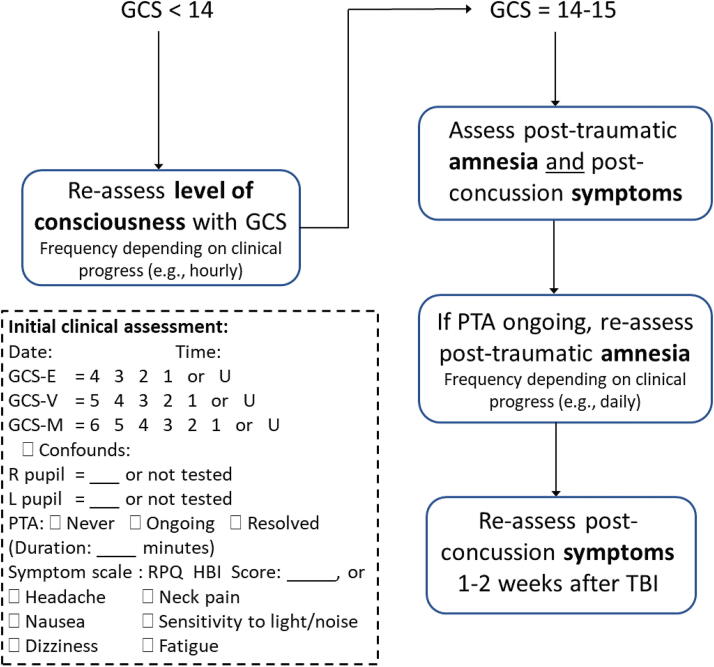
Recommended algorithm for incorporation of pupil, PTA, and symptom assessment into TBI clinical care pathways. The dash-outlined box provides an example documentation template. PTA, post-traumatic amnesia; TBI, traumatic brain injury.

Initial assessment of PTA is not always necessary or feasible in the emergency setting. Patients with clear evidence of confusion or impaired consciousness need not be assessed with a PTA scale (see Fig. 2), but rather only those with GCS = 15 or GCS = 14 where documentation of confused behavior is absent or equivocal. Feasibility issues include insufficient time and prominent confounding factors, e.g., opiate analgesic medications can impair episodic memory and lower performance in the Westmead PTA Scale.^43,44^

Finding that a patient has ongoing PTA in the ED may have implications for clinical decision-making, such as whether to order a head CT,^45^ move the patient to a secure and low-stimulation environment,^41^ or discharge them home with supervision or admit to hospital for observation.^24^ If a patient is still in PTA (or coma), serial PTA testing after transfer from the ED will be necessary to accurately monitor emergence from PTA and quantify its duration.^41^ Prospective serial assessment is important because retrospective estimation of PTA months after the injury is unreliable.^46^ That said, it may be more useful than no information about PTA.

Many patients will have experienced a period of PTA that resolved prior to their arrival at the ED. If a patient is confirmed to not be in PTA on ED assessment, the clinician should query for resolved PTA by asking the patient if they remember the impact and moments after, what their first memory following the impact is, and when their memories became continuous again, suggesting emergence from PTA. Subtracting the time of emergence from PTA from time of injury can provide a crude estimate of PTA duration. A crude estimate may be sufficient in this context because although PTA duration in the range for 1–60 days is highly prognostic, PTA duration within the range of 0–24 h appears less so.^37,38^ Another rationale for querying pre-hospital arrival PTA is that can help rule-in a diagnosis of TBI in a patient without other acute signs.^15^

In summary, the CSWG recommend that patients with a GCS of 14–15 should have the presence of PTA assessed and the resulting information be incorporated into clinical care pathways as suggested in Figure 2.

#### Mechanisms of injury

Low energy transfer mechanisms (e.g., falls from a standing height or <2 m in adults) are conventionally expected to result in less severe injury than high-velocity injury (e.g., road traffic collisions, falls from a greater height), and information on injury mechanisms should be routinely recorded.^24^ However, even low energy transfer incidents (especially falls in infants and older patients) can cause significant injury that is under-estimated by conventional clinical assessment, results in under-triage and inadequate or delayed investigation or treatment.^47^ Assessment of the mechanism of injury is usefully supplemented by data regarding protective measures (seat belts, airbags, helmets) that might mitigate the injury, since these can substantially reduce the extent of injury.^48^

#### TBI symptoms

Traditional indicators of neurological status such as GCS exhibit minimal variability and ceiling effects, and have limited utility in the large proportion of patients with TBI who present with a GCS of 15, have emerged from PTA, and have a normal CT. Prospective cohort studies in non-hospitalized TBI have considered a broader range of clinical variables available in the ED and found that most have limited prognostic value.^38,49–51^ ED assessment of symptoms (e.g., headache, dizziness, sensitivity to noise) using validated scales may be an exception, with some evidence in both children^52,53^ and adults,^51,54,55^ for age-appropriate scales. Relevant instruments include the Rivermead Post Concussion Symptoms Questionnaire for adults; and the Health Behavior Inventory (the symptom scale embedded in the Child Sport Concussion Assessment Tool 6) for children. There is also more robust evidence for the prognostic utility of acute symptom assessment following sport-related concussion,^56^ which could be reasonably extrapolated. The evidence that symptoms are prognostic primarily applies to patients with a GCS of 15 (or a verbal score of 5)—but it is reasonable to record symptoms in patients with a GCS verbal score of 4. The evidence for presence/absence of specific symptoms in ED is mixed.^51,52,54,55,57^ Symptom severity ratings may be more prognostic than symptom presence/absence,^58^ perhaps because they contain more granular information and have more variability. Symptom reporting in the 7–14 days following injury has been consistently shown to improve prognostic accuracy in non-hospitalized TBI over and above demographic and clinical variables,^37,50,51,54^ and acute symptoms measured in the ED.^58,59^ For this reason, the clinical assessment algorithm (Fig. 2) recommends both acute and follow-up symptom assessments. More broadly, the variable prognostic utility of symptoms provides a strong argument for integrating biofluid biomarker and neuroimaging data into a comprehensive assessment of TBI.

Studies that evaluate the prognostic utility of post-concussion symptoms are summarized in Supplementary Table S1a and b. These studies used varied symptom lists, administration modalities (e.g., interview vs. self-report), response options, and scoring methods (e.g., symptom count vs. summed item severity ratings), between 0 and 20 days following injury. Few of these studies included pediatric patients, and there is almost no literature relevant to the relationship of symptoms and outcomes in children <5 years of age. All studies reported strong associations with outcome, as measured by the Glasgow Outcome Scale-Extended or the presence of multiple persistent symptoms at 1–6 months follow-up.

#### Objective assessment of cognition, balance, and vestibulo-oculomotor dysfunction

There is emerging evidence that assessing cognition with standardized objective tests in the ED or soon after may further refine the prognosis of non-hospitalized TBI.^60–66^ CSWG found inadequate evidence to support the prognostic utility of performance-based clinical assessment tools after non-hospitalized TBI. Standardized objective tests of cognition have been most studied for prognostic utility. They have demonstrated significant associations with outcome in multiple studies.^60–67^ However, most studies used different cognitive tests and involved small samples, without confound adjustment or external validation. Because objective cognitive tests have prognostic utility in hospitalized TBI,^68–70^ they have the potential to contribute to the classification of TBI across the severity spectrum. Other performance-based clinical assessment tools such as the King-Devick Test,^71–73^ Vestibular/Oculomotor Screening Test,^73–77^ Buffalo Concussion Treadmill Test,^78,79^ EyeBox,^80^ and various measures of standing balance or gait^81–83^ have been explored for their prognostic utility in various settings but their relative prognostic utility at different time points after injury has not been established. Moreover, findings from these studies are inconsistent and may not be representative as they are almost exclusively based on studies with athletes who sustained a sport-related concussion, a narrow segment of the non-hospitalized TBI population. Importantly, it is as yet unclear if any performance-based clinical assessment tools can predict outcome over and above symptom severity ratings, which are more feasibly obtained.

### Additional clinical evaluations for specific populations

In specific populations, additional clinical evaluations should be considered. For example, the U.S. Department of Defense recommends the use of the Military Acute Concussion Evaluation version 2 (MACE 2) in service members with suspected TBI, GCS 13–15, and no “red flags” concerning more serious injury (https://health.mil/Reference-Center/Publications/2020/07/30/Military-Acute-Concussion-Evaluation-MACE-2). The MACE 2 includes a brief, standardized neurological examination that can be performed consistently by providers with a wide range of training and experience (PMID: 32808563). It also includes the Vestibulo/Ocular-Motor Screening (VOMS) recommended for those who are not overtly symptomatic at baseline and do not have unstable cervical spine. The VOMS consists of a series of provocative maneuvers that may bring out otherwise occult concussion-related symptoms.^84^ The scores on the MACE 2 including symptoms provoked by VOMS can be tracked serially over time to measure recovery, guide progressive return to activity, and assist with return-to-duty decision-making (https://health.mil/Reference-Center/Publications/2024/02/23/Progressive-Return-to-Activity-Primary-Care-for-Acute-Concussion-Management). However, evidence for the longer-term prognostic utility of the MACE 2 is inconsistent at present.^59,85^

#### Extracranial injury

Compared with isolated TBI, polytrauma is associated with a higher risk of moderate disability and severe disability/death, at both 3 and 6 months.^86,87^ These worse outcomes may be due to the injury itself, a higher risk of early hypoxia and hypotension,^88^ an aggravated detrimental host response,^89^ and/or the effects of anesthesia and surgery needed for extracranial injuries.^90^ These considerations mandate a systems-based tertiary trauma assessment in all patients with TBI. A range of trauma severity assessment tools have been used in this context, but the Abbreviated Injury Score (AIS)^91^ is probably most widely used. Both the head AIS and the Injury Severity Score (the sum squares of AIS scores in the three most severely injured regions) may be of some prognostic value in TBI,^92^ but an AIS ≥3 in any individual extracranial region also provides a convenient and pragmatic threshold for identifying extracranial injuries that are of relevance in the integrated characterization of multiple trauma that includes TBI, in registries and research studies.^86^ If a formal assessment of AIS is thought to be less practicable for routine clinical evaluation, a useful approximation may be to record any injury that would, in isolation, have required hospital admission.^86,87^ Characterization of the severity of extracranial injuries is included in the Modifier Pillar of the CBI-M model. Such an integrated assessment of the severity of TBI and extracranial injury provides the best basis to plan extracranial surgery (balancing the risks and benefits of early definitive treatment against the risks of perioperative physiological compromise in a vulnerable brain). Such an assessment also allows for rational planning of follow-up and rehabilitation.

#### Early physiological insults and seizures

Hypoxia, hypotension, hypothermia, and fever at presentation have all been associated with worse outcomes in TBI, and their presence should be recorded in any complete clinical characterization of TBI. However, the most appropriate thresholds for identifying these insults are still not clear and the field is still evolving. For example, Traumatic Coma Data Bank (TCDB) data suggested a systolic blood pressure (SBP) threshold of 90 mmHg,^93^ but more recent publications suggest a higher thresholds,^94^ or a U-shaped association with outcome^95^ Similarly, while TCDB data focused on hypoxia,^60^ there is increasing exploration of hyperoxia as a risk factor,^96^ and early (spontaneously achieved) peak temperatures below 37°C or above 39°C are associated with worse outcomes.^97^ Despite these epidemiological associations, precise thresholds for defining harmful levels of blood pressure, oxygen saturation, and temperature remain unclear, as do the indications and means for treating these. Consensus-based thresholds (such as those identified by the American College of Surgeons Trauma Quality Improvement Program Guidelines^98^) may be useful to define hypoxia and hyperoxia, hypotension and hypertension, and hypothermia and fever, until definitive data emerge on this. While not discussed here, it is also important to recognize that blood pressure norms (and by extension, harm thresholds and clinical targets) vary substantially with age,^99–102^ a consideration that is critical in managing pediatric TBI. While hypoglycaemia, hyperglycaemia, and hyponatremia represent additional important metabolic insults^98^ and are often available at the time of ED assessment in patients, they are not part of clinical assessment, and are hence not covered here.

Early post-traumatic seizures have been independently associated with increased need for ICU admission, longer hospital stay, dependency at discharge, and worse functional outcome.^103^ It is important to record the presence of early post-traumatic seizures not only because of these associations but also because a post-ictal state may be responsible for impairment of consciousness, and provide a reason for caution against estimating TBI severity simply based on the GCS.

#### Age, comorbidities, and frailty

Age is among the strongest outcome predictors in TBI, with mortality and unfavorable outcome increasing continuously with age through adulthood.^104,105^ This may be due to reduced physical or neurological reserve, and/or the presence of comorbid disease, which is often (though not exclusively) associated with aging. The exception of these trends is in children, where infants have a higher mortality rate than older children,^106^ and other outcomes have complex relationships with age.^107^ While such knowledge should inform how clinicians counsel patients and families about prognosis and the benefits of aggressive therapy, it is important to avoid a nihilistic response to TBI management in all older patients, since such nihilism may (in itself) contribute to inconsistent WLST^25,26^ and poor outcomes.^108^ Indeed, even in an ICU setting, a significant proportion of such older patients may achieve a favorable recovery with appropriate therapy.^109^ More refined approaches are needed to assess the impact of age and pre-existing disease.

One key approach is to refine age-related vulnerability by recording frailty, a term used in both adults and children, which quantifies loss of physiological and cognitive reserve, and may increase vulnerability to the stress of trauma. Additional Frailty scales may be based on the presence of comorbidities (such as the Charlson Comorbidity Index [CCI], which can be reliably abstracted from electronic patient records^110^; the 70-item Canadian Study of Health and Aging [CSHA] Frailty Index^111^; the modified 5- and 11-item Frailty Index [mFI-5 and mFI-11]^112^; and a five-item Pediatric Frailty Scale).^113^ The mFI-5 and mFI-11 are associated with worse outcome in TBI,^114,115^ and a novel 30-item scale was also associated with worse TBI outcome in the CENTER-TBI and TRACK-TBI studies.^116^ While these scales clearly have research relevance, they may be difficult to implement in practice. Global clinical assessments, such as the CSHA Clinical Frailty Scale (CFS),^111^ associate with the outcome, with threshold scores of ≥4^117^ or ≥3^118^ on the 9-point CFS associated with a ∼90–95% risk of death or severe disability. The CFS may provide a more pragmatic option for recording frailty in the context of clinical TBI management.

The discussion above primarily focuses on physical comorbidities and systemic physiological reserve, both of which have been shown to be important in modulating TBI outcome.^119^ These scales also address pre-injury neurological status but only in the context of established diagnoses. Current assessments do not address cognitive reserve or psychological health—both of which can be critical determinants of TBI outcome (and are covered by another Working Group). There is a need for better means of quantifying the impact of these factors.

Other considerations apply at the younger end of the age spectrum. In young children, early recovery may be excellent, but children who sustain a TBI and appear to recover fully may be on a different developmental trajectory from their uninjured peers, and disabilities may only manifest years after the injury.^120^ It is unclear whether initial assessment tools can identify children most at risk of such adverse late outcomes, and research in this area is needed.

A separate article in this series^4^ provides further consideration of Psychosocial and Environmental “Modifiers” that can impact outcome (independent of injury severity) or influence assessment.

#### Concurrent therapy

It is critical that a full characterization of acute TBI also records pre-injury therapies that the patient is receiving, with particular attention to medication that may affect the disease course in TBI. While several drugs may be relevant in this context, anticoagulants and antiplatelet agents have a direct impact on hematoma expansion and outcome, and are most widely addressed in the literature.^121^

#### Additional information over the first 2 weeks post-injury

TBI pathophysiology evolves over time, and incorporating additional clinical information over the initial course provides an improved selection of patients for acute therapy and follow-up and refines late (months to years) prognostication. The ways in which such dynamic information is collected will depend on TBI severity and care path.

##### For non-hospitalized TBI

Assessing post-TBI symptom severity (using the Rivermead Post Concussion Symptoms questionnaire or comparable instruments) up to 14 days after injury has been repeatedly shown to refine prognosis,^37,51,54^ likely above and beyond acute symptom severity. Several studies additionally measured mental health symptoms using validated self-report scales, designed to quantify symptoms of depression (e.g., PHQ-9),^122^ anxiety (e.g., GAD-7),^123^ and/or post-traumatic stress (e.g., PCL-5),^124^ and found that these scales explained unique variance in outcome from non-hospitalized TBI.^55,57,125^ Symptom assessment could also inform the need for repeat biomarkers, further follow-up, MRI, or inclusion in trials (Fig. 1). For logistic reasons, attempts have been made to identify, at presentation, patients particularly high risk of persistent symptoms for such follow-up,^51^ but this remains an imperfect process, and an area in which future research should be prioritized.

*For hospitalized TBI*, ongoing assessment of neurological status, intracranial and systemic physiology, and therapy requirements provides important information for characterizing TBI and informing prognostication. Specific items include:

• Clinical neuroworsening (drop in GCS, seizures, progression of neurological deficit, development of a new neurological deficit, or new pupillary abnormality) is important for both prognosis and therapy.^126^
• Monitoring of systemic physiology, intracranial pressure, brain oxygenation and metabolism, and electrophysiology (which may allow detection of non-convulsive seizures); and charting of therapy intensity level and response.^127,128^ It is likely that, in the future, these complex data can be usefully integrated and synthesized using novel data science approaches (including machine learning and artificial intelligence) to provide decision support tools that allow more individualized and precise management and prognostication. These can either cover the entire disease narrative of TBI for general prognosis of outcomes,^129–131^ or address more specific contexts, such as prediction of intracranial hypertension,^132–134^ emergence from coma,^135^ and predicting benefit from rehabilitation.^136^ However, the clinical use of such tools is still being developed, and no validated applications are currently available. Consequently, this is best considered an important area for research, rather than a recommendation for routine clinical use.
• Daily assessment of post-traumatic amnesia, using the GOAT, Westmead Post Traumatic Amnesia (WPTA) Scale, or O-Log^41^ in the period of emergence following hospitalized TBI can improve prognostication over and above the initial GCS score.^137–139^

## Conclusions

The CSWG assessed the features available for characterization of patients following TBI based on their value in informing prognostication and informing clinical decision-making and clinical care in the acute care hospital setting. Key targets included appropriate triage, accurate diagnosis of TBI, optimized resuscitation targets to minimize secondary injury, triggers for diagnostic interventions (such as blood biomarkers and neuroimaging), allocation of patients to clinical pathways, and identification of follow-up needs. The recommendations of the CSWG are summarized in Table 1.

**Table 1.** Recommendations of the Clinical/Symptoms Working Group: Clinical Characterization of TBI <24 h Post-Injury^a^

(1) Basic clinical descriptors
For all patients, the following must be recorded:
Glasgow Coma Scale (GCS) (full breakdown: motor (M), verbal (V), and eye (E) components).
• Use post-resuscitation GCS for consistency.
• Explicitly note confounds (e.g., intoxication, sedation, intubation).
• Untestable GCS components should be marked with the suffix “U” (untestable) and scored as 1 in sum GCS^b^
Pupillary responses:
• Report independently from GCS, but assess at the same time as GCS.
• Avoid using an integrated GCS-P score.
• Use automated pupillometry when possible.
(2) Expanded clinical characterization
For a more complete TBI assessment, record:
Injury factors:
• Mechanism, impact velocity, and mitigation (e.g., seat belts, airbags, helmets).
• Extracranial injuries that would warrant hospital admission, even in the absence of a TBI
• Early physiological insults (to include hypoxia and hypotension; based on TQIP consensus thresholds)
• History of loss of consciousness (LoC)
• Presence and duration of post-traumatic amnesia (PTA) duration, ideally determined by prospective serial assessment with a validated tool. Record assessment point (e.g., arrival to trauma ward) and time post-injury
• In patients with GCS verbal score >4 in ED: document acute symptoms, ideally with standardized rating scales
Biopsychosocial-ecological vulnerabilities:
• Physical/psychological comorbidities.
• Relevant therapies (especially those affecting hemostasis).
• Age, frailty, socioeconomic status, education, and employment status.
Dynamic assessment:
• Record neuroworsening (GCS, pupillary reactivity, neurological examination) over the first 7–14 days.
• Monitor symptom severity over first 7–14 days in patients not admitted to the hospital.
(3) Emerging clinical variables
Consider these additional assessments, though further validation is needed of their use and utility:
• Vestibulo-oculomotor dysfunction and balance, particularly for less severe cases.
• Cognitive assessment: standardized objective tests soon after injury (no specific platform recommended).
• Mental health: assess symptoms 7–14 days post-injury using validated scales.
Research recommendations
• Address empirical validation, refinement, implementation, and impact of the recommendations listed above.
• Define optimal approaches for assessment and notation where components of the GCS are not assessable
• Define the objective and widely accepted thresholds to characterize the full range of physiological insults.
• Evaluate data-driven tools that integrate dynamic and imputed data for prognostication and decision support.

^a^ These recommendations apply to patients presenting to hospital <24 h post-injury, with features recorded as part of a clinical, biomarker, imaging (CBI) framework, as well as recording modifiers (M) that may affect assessment or modify expected outcomes. Items in the clinical (C) pillar of CBI-M are classified into three categories, with a separate listing of research recommendations.

^b^ Untestable: e.g., M_U_ V_U_ E_U_ = GCS: 3U for a patient who is sedated and intubated.

TBI, traumatic brain injury; TQIP, trauma quality improvement program.

The CSWG concluded that the GCS continues to provide an excellent clinical basis for TBI characterization of patients with TBI. However, current trichotomized categorization of TBI as mild/moderate/severe is not fit for purpose, and can lead to imprecise prognostication and inappropriate clinical management.

Appropriate use of the GCS requires recording of the full GCS sum score (ideally post-resuscitation), with breakdown of its eye, verbal, and motor components to allow appropriate characterization of patients. There needs to be explicit identification of confounds to assessment (e.g., alcohol or drug effects) and untestable components (as “U”; e.g., due to endotracheal intubation). However, the optimal approaches to assessing and recording GCS in such settings need further work. All patients should have pupillary reactivity to light in both eyes assessed and recorded separately from the GCS. The GCS has ceiling and floor effects, and in patients with a sum GCS score of 14/15, the Working Group recommended recording a history of LoC and the presence and duration of post-traumatic amnesia (PTA) using validated tools. In patients with a verbal GCS score ≥4, acute symptoms should be documented, using standardized rating scales appropriate for the context of use.

Other key variables include injury factors (mechanism, injury velocity, and impact mitigation factors) and acute physiological insults (based on expert consensus thresholds). A more complete characterization of TBI should also include biopsychosocial-ecological vulnerabilities: comorbidities, concurrent therapies, assessment of frailty in addition to age (using validated instruments), socioeconomic status, educational attainment, and employment status. Finally, the CSWG recognized the importance of recording disease progression or resolution over the first 14 days—including neuroworsening in hospitalized patients, and serial assessment of symptom severity in all patients.

Additional assessments that have emerging (but as yet inconclusive) evidence for use include a detailed assessment of neurological deficits, vestibulo-oculomotor dysfunction, cognition (using standardized tests), and assessment of mental health symptoms. Data-driven integration of physiological status and therapy intensity could, in the future, provide decision support tools in hospitalized patients, but these require further refinement, validation, and implementation.

This article provides recommendations for clinical assessments as part of an integrated CBI-M scheme for patient assessment. Routine assessment of these clinical features, alongside blood-biased biomarkers, neuroimaging, and psychosocial/environmental modifiers, can refine TBI characterization and potentially improve injury outcomes. However, it is critical to recognize that high-quality evidence for the use of the variables addressed in this article is limited. Consequently, these recommendations are based on expert consensus. Additional research is needed to validate the use of these recommendations, both individually, and as part of an integrated CBI-M scheme. Such research must address performance of the scheme in achieving better prognostic precision, and in improving clinical decision-making and care. However, it is also critically important that such evaluation also addresses issues in appropriate implementation of these recommendations, so as to establish robust links between knowledge and practice.
