# Supplementary material for: Clinical Assessment on Days 1–14 for the Characterization of Traumatic Brain Injury: Recommendations from the 2024 NINDS Traumatic Brain Injury Classification and Nomenclature Initiative Clinical/Symptoms Working Group
Source: J Neurotrauma. 2025 Jul 9;42(13-14):1038–55. doi: 10.1089/neu.2024.0577 (PMC12417841; doi:10.1089/neu.2024.0577)
Supplement: Supplementary Data [file neu.2024.0577_supplementary_data.docx]

**Supplementary material**

**Clinical Assessment on days 1 – 14 for the characterisation of traumatic brain injury: Recommendations from the** **2024 NINDS Traumatic Brain Injury Classification and Nomenclature Initiative Clinical/Symptoms Working Group**

David K Menon, Noah D Silverberg, Adam R Ferguson, Thomas J Bayuk, Shubhayu Bhattacharyay, David L Brody, Scott A Cota, Ari Ercole, Anthony Figaji, Guoyi Gao, Christopher C Giza, Fiona Lecky, Rebekah Mannix, Ana Mikolić, Kasey E Moritz, Claudia S Robertson, Abel Torres-Espin, Spyridoula Tsetsou, John K Yue, Hibah O Awwad HO, Kristen Dams-O’Connor, Adele Doperalski, Andre IR Maas, Michael A McCrea, Nsini Umoh, Geoff T Manley.

**Supplementary material Page**

| **Membership of the Clinical/Symptoms Assessment Working Group** | **3** |
| --- | --- |
|  |  |
|  |  |
|  |  |
|  |  |

**Membership of the Clinical/Symptoms Assessment Working Group**

*Workgroup Chairs*

Adam R Ferguson (University of California San Francisco; [Adam.Ferguson@ucsf.edu](mailto:Adam.Ferguson@ucsf.edu))

David K Menon (University of Cambridge; [dkm13@cam.ac.uk](mailto:dkm13@cam.ac.uk))

Noah D Silverberg (University of British Columbia; [noah.silverberg@ubc.ca](mailto:noah.silverberg@ubc.ca))

*Workgroup Members*

Thomas J Bayuk (Uniformed Services of the Health Sciences; [thomas.bayuk@usuhs.edu](mailto:thomas.bayuk@usuhs.edu))

Matt Breiding (Centers for Disease Control and Prevention; [dvi8@cdc.gov](mailto:dvi8@cdc.gov))

David L Brody (Uniformed Services University of the Health Sciences; [david.brody@usuhs.edu](mailto:david.brody@usuhs.edu))

Todd A Cesar (TBICoE DHA; [todd.a.cesar.mil@health.mil](mailto:todd.a.cesar.mil@health.mil))

Scott A Cota (TBICoE DHA; [scott.a.cota.mil@health.mil](mailto:scott.a.cota.mil@health.mil))

Ari Ercole (University of Cambridge; [ae105@cam.ac.uk](mailto:ae105@cam.ac.uk))

Anthony Figaji (University of Cape Town; [Anthony.Figaji@uct.ac.za](mailto:Anthony.Figaji@uct.ac.za))

Guoyi Gao (Capital Medical University, Beijing; [guoyigao@gmail.com](mailto:guoyigao@gmail.com))

Christopher Giza (University of California Los Angeles; [cgiza@mednet.ucla.edu](mailto:cgiza@mednet.ucla.edu))

Fiona Lecky (University of Sheffield; [f.e.lecky@sheffield.ac.uk](mailto:f.e.lecky@sheffield.ac.uk))

Rebekah Mannix (Boston Children’s Hospital; [Rebekah.Mannix@childrens.harvard.edu](mailto:Rebekah.Mannix@childrens.harvard.edu))

Kasey Moritz (Combat Casualty Care Research Program; [kasey.e.moritz.civ@health.mil](mailto:kasey.e.moritz.civ@health.mil))

Claudia S Robertson (Baylor College of Medicine; [claudiar@bcm.tmc.edu](mailto:claudiar@bcm.tmc.edu))

John Yue (University of California San Francisco; [yuej@neurosurg.ucsf.edu](mailto:yuej@neurosurg.ucsf.edu))

*Affiliate Members*

Shubhayu Bhattacharyay (University of Cambridge; [sb2406@cam.ac.uk](mailto:sb2406@cam.ac.uk))

Ana Mikolic (University of British Columbia; [ana.mikolic@ubc.ca](mailto:ana.mikolic@ubc.ca))

Abel Torres-Espin (University of Waterloo; [abel.torresespin@uwaterloo.ca](mailto:abel.torresespin@uwaterloo.ca))

Spyridoula Tsetsou (Baylor College of Medicine; [Spyridoula.Tsetsou@bcm.edu](mailto:Spyridoula.Tsetsou@bcm.edu))

**Transparency, Rigor, and Reproducibility Statement**

The scope of the work in this manuscript was based on a pragmatic decision by the Working Group Chairs (DKM, NDS, ARF) in discussion with the NINDS TBI Classification and Nomenclature Workshop Steering Committee. We recognise that a large proportion of patients who sustain a TBI never present to hospital, but for pragmatic reasons, we limited our scope to TBI in patients presenting to hospital. Similarly, we recognise that TBI is a globally leading cause of death and disability for children, but a detailed discussion of pediatric TBI was precluded by practical considerations. We did not undertake fresh formal systematic reviews of the topics covered in this manuscript – our work was informed by expert pragmatic reviews of the literature, led by one to three of the Working Group members with specific expertise on the topic, followed by detailed discussions of their evidence summaries in the Working Group meetings. This synthesis of evidence drew on the experience of working group members in authoring/co-authoring the recent NASEM Report,^a^ authoritative summative reviews,^b,c^ and clinical guidelines,^d,e,f^ as well as the outputs of InTBIR studies (<https://intbir.incf.org/>). This manuscript was informed by discussions at the NINDS TBI Classification and Nomenclature Workshop (<https://www.ninds.nih.gov/news-events/events/ninds-tbi-classification-and-nomenclature-workshop>) and by the powerful personal testimony of individuals with lived experience of TBI. Where appropriate, we also incorporated evidence that became available after the workshop – specifically addressing the use of the GCS-P score^g^ (reference 32 in the manuscript). The recommendations that we make on clinical assessment must be integrated with the wider Clinical, Biomarker, Imaging and Modifier (CBI-M) framework recommended by the Workshop process and supported by publications from the other Working Groups involved in the Workshop. We need to acknowledge that our recommendations are based primarily on expert interpretation of the literature and rely on associations of clinical features with outcome, rather than empirical evidence. Consequently, these recommendations require formal validation and refinement in future studies, and formal assessments of implementability so as to underpin the transition from knowledge to practice.

**References**

1. Traumatic Brain Injury: A Roadmap for Accelerating Progress (<https://nap.nationalacademies.org/login.php?record_id=25394>).
2. Maas AIR, Menon DK, Adelson PD, et al; InTBIR Participants and Investigators. Traumatic brain injury: integrated approaches to improve prevention, clinical care, and research. Lancet Neurol. 2017 Dec;16(12):987-1048. doi: 10.1016/S1474-4422(17)30371-X. Epub 2017 Nov 6. PMID: 29122524.
3. Maas AIR, Menon DK, Manley GT, et al; InTBIR Participants and Investigators. Traumatic brain injury: progress and challenges in prevention, clinical care, and research. Lancet Neurol. 2022 Nov;21(11):1004-1060. doi: 10.1016/S1474-4422(22)00309-X. Epub 2022 Sep 29. Erratum in: Lancet Neurol. 2022 Dec;21(12):e10. doi: 10.1016/S1474-4422(22)00411-2. PMID: 36183712; PMCID: PMC10427240.
4. ACS TQIP BEST PRACTICES IN THE MANAGEMENT OF TRAUMATIC BRAIN INJURY. (<https://www.facs.org/media/mkej5u3b/tbi_guidelines.pdf>)
5. Head injury: assessment and early management: NICE guideline [NG232] <https://www.nice.org.uk/guidance/ng232>
6. Silverberg ND, Iverson GL; ACRM Brain Injury Special Interest Group Mild TBI Task Force members. The American Congress of Rehabilitation Medicine Diagnostic Criteria for Mild Traumatic Brain Injury. Arch Phys Med Rehabil. 2023 Aug;104(8):1343-1355. doi: 10.1016/j.apmr.2023.03.036. Epub 2023 May 19. PMID: 37211140.
7. Vreeburg RJG, Leeuwen FD van, Manley GT, et al. Validation of the GCS-Pupil Scale in Traumatic Brain Injury Incremental Prognostic Performance of Pupillary Reactivity with GCS in the Prospective Observational Cohorts CENTER-TBI and TRACK-TBI. 2024;2024.06.05.24308424; doi: 10.1101/2024.06.05.24308424.
