# Supplementary material for: Clinical Assessment on Days 1–14 for the Characterization of Traumatic Brain Injury: Recommendations from the 2024 NINDS Traumatic Brain Injury Classification and Nomenclature Initiative Clinical/Symptoms Working Group
Source: J Neurotrauma. 2025 Jul 9;42(13-14):1038–55. doi: 10.1089/neu.2024.0577 (PMC12417841; doi:10.1089/neu.2024.0577)
Supplement: Supplementary Figure S1 [file neu.2024.0577_supplementary_figures1.docx]

**Supplementary Figure 1.**

Overall predictive performance of different regression models in 4960 patients in the combined CENTER-TBI (n = 3521) and TRACK-TBI (n = 1439) datasets. Mean and 95% confidence intervals for Nagelkerke’s R^2^ for mortality (in blue) and unfavorable outcome (in green) for GCS, combined GCS-P score (as described in Brennan et al^31^), and addition of GCS and pupillary reactivity calculated separately. Estimates and 95% CI are bootstrapped. ΔR^2^ values are differences from the baseline model containing only GCS (labelled as ΔR^2^: 0) Drawn from data in Vreeburg et al, 2024**.**^32^

**
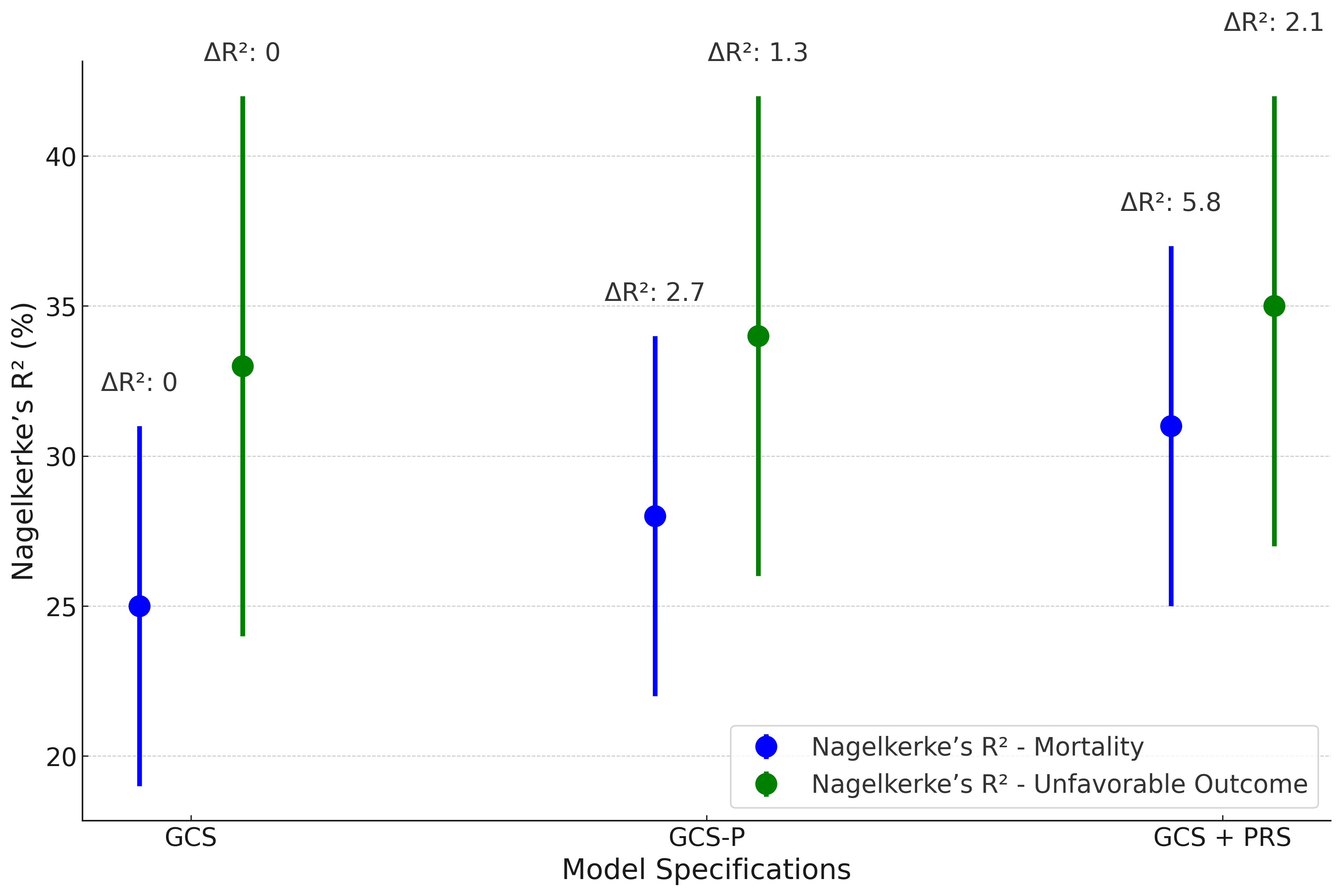
**
