# Supplementary material for: Clinical Assessment on Days 1–14 for the Characterization of Traumatic Brain Injury: Recommendations from the 2024 NINDS Traumatic Brain Injury Classification and Nomenclature Initiative Clinical/Symptoms Working Group
Source: J Neurotrauma. 2025 Jul 9;42(13-14):1038–55. doi: 10.1089/neu.2024.0577 (PMC12417841; doi:10.1089/neu.2024.0577)
Supplement: Supplementary Figure S2 [file neu.2024.0577_supplementary_figures2.docx]

**Supplementary Figure 2:**

Mortality and unfavorable outcome distribution at six months post-TBI for GCS-P score 3 (n = 397), separated by GCS-components and PRS from combined TRACK-TBI and CENTER-TBI data. Data on left are from patients with GCS score E1V1M1 and both reactive pupils (n = 381); in middle from patients with GCS score E1V1M2 and one underactive pupil (n = 5); and on right from patients with GCS score E1V1M2 and both pupils unreactive (n = 11). There are significant differences in mortality (p <0.01; χ^2^ test statistic: 9.2) and unfavorable outcome (p <0.01; χ^2^ test statistic: 10) between the three groups. Drawn from data in Vreeburg et al 2024.^32^

**
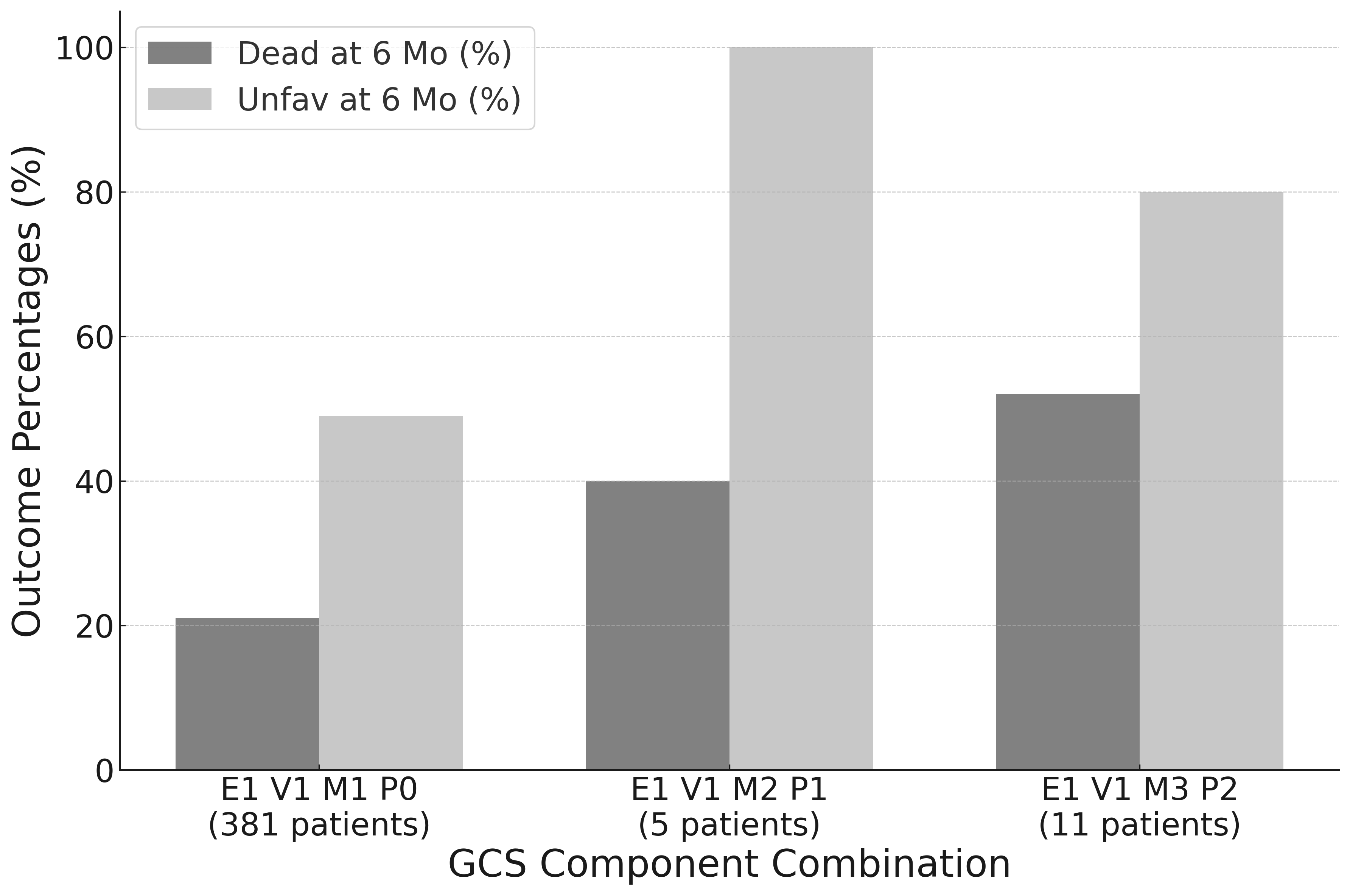
**
