# Supplementary material for: Clinical Assessment on Days 1–14 for the Characterization of Traumatic Brain Injury: Recommendations from the 2024 NINDS Traumatic Brain Injury Classification and Nomenclature Initiative Clinical/Symptoms Working Group
Source: J Neurotrauma. 2025 Jul 9;42(13-14):1038–55. doi: 10.1089/neu.2024.0577 (PMC12417841; doi:10.1089/neu.2024.0577)
Supplement: Supplementary Table S1 [file neu.2024.0577_supplementary_tables1.docx]

**Supplementary Table 1a:** Overview of prediction models that include early symptoms (1-3 weeks) for predicting persistent symptoms (PPCS) and functioning (Glasgow Outcome Scale Extended, GOSE) after mild traumatic brain injury

| **First author, year** | **Study, country** | **Population, sample size** | **Early symptoms,**  **Measurement**  **, Timing** | **Odds ratios (OR) or regression coefficients, 95% Confidence intervals (CI)** | **Other predictors** | **Outcome** | **Performance** |
| --- | --- | --- | --- | --- | --- | --- | --- |
| Stulemeijer,^54^  2008,  Rule | RUBICS.  The Netherlands | GCS 13-15, LOC<30 min,  18-60 years,  Level 1 trauma center;  N=201 | Post-concussion symptoms (PCS): RPQ;  Post-traumatic stress: IES  0-37 days, median 9 days | Severe PCS on RPQ: OR 5.5, 95% CI 2.3 to 13.2;  IES total score >26: OR 5.5, 95% CI 2.3 to 13.2 | Pre-injury physical health | 6-month PPCS  (RPQ) | AUC=0.73 |
| Van der Naalt, ^53^2017  Model | UPFRONT,  The Netherlands | GCS 13-15, LOC<30 min, PTA<24h,  Adult,  Level 1 trauma centers;  N=671 | PCS (Head Injury Severity Checklist, HISC),  Depression (HADS), Anxiety (HADS), Passive coping style (Utrecht Coping List, UCL), Avoidant coping style (UCL);  2 weeks | HISC: OR* 0.57, 95% CI 0.40 to 0.81;  HADS Depression: OR* 0.44, 95% CI 0.33 to 0.59;  HADS Anxiety: OR* 1.54, 95% CI 1.07 to 2.22;  UCL- Passive: OR* 0.65, 95% CI 0.42 to 1.02;  UCL-Active: OR* 1.29, 95% 1.02 to 1.63 | Education, Mental health history, Alcohol intoxication, Neck pain, GCS, PTA duration | 6-month  GOSE=8 | AUC=0.77 |
| Cnossen,^56^  2018,  Model | UPFRONT,  The Netherlands | GCS 13-15 with LOC/PTA,  Adult,  Level 1 trauma centers;  N=591 | PCS (HISC);  Post-traumatic stress (IES);  2 weeks | PCS based on ICD-10: OR 4.89, 95% CI 3.19 to 7.49;  IES total score >26: OR 2.98, 95% CI 1.88 to 4.73 | Sex,  Neck pain, Nausea,  Headache | 6-month PPCS  (HISC) | AUC=0.75 |
| Le Sage, ^50^  2022,  Rule | PoCS, Canada | GCS 13-15, LOC<30 min, PTA<24h,  Age >=14 years,  Level 1 and 2 trauma centers;  N=471 | PCS (RPQ);  7 days | RPQ Headaches >=2: OR 3.14, 95% CI 1.27 to 7.76  RPQ Sleep disturbance >=2: OR 1.74, 95% CI 0.83 to 3.66  RPQ Fatigue >=2: OR 2.09, 95% CI 0.76 to 5.74;  RPQ Light sensitivity >=2: OR 2.67, 95% CI 1.29 to 5.53;  RPQ total score >=21: OR 3.24, 95% CI 1.45 to 7.24. | Age, Sex,  Prior multiple TBI, prior TBI <year, Mental health history,  Cervical sprain, Hemorrhage on CT | 3-month PPCS (RPQ) | AUC=0.85 for follow-up assessment. Rule: Sensitivity 0.92 and specificity 0.54. |
| Mikolic,^37^  2023  Model | CENTER-TBI, Europe | GCS 13-15,  16+years,  Level 1 trauma centers; subset mainly discharged home from ED  N=476 | PCS (RPQ),  Post-traumatic stress (PCL-5),  Anxiety (GAD-7);  2-3 weeks (median 20 days) | RPQ total score*: 7.82, 95% CI 5.98 to 9.66.  PCL-5 total score*: 1.79, 95% CI -0.04 to 3.61.  GAD-7 total score*: 1.85, 95% CI -0.34 to 4.03 | Sex, Mental health history,  Preinjury health, Injury severity score | 6-month PPCS (RPQ) | R2=0.37 for RPQ total score;  C=0.83 for RPQ>=16 |
|  |  | N=640 | PCS (RPQ),  Post-traumatic stress (PCL-5);  2-3 weeks (median 20 days) | RPQ total score: OR* 0.37, 95% CI 0.26-0.52  PCL-5 total score: OR* 0.71, 95% CI 0.55-0.91 | Injury severity score, Age, GCS, Mental health history, Cause of injury | 6-month GOSE  (1-8) | Nagelkerke R2=0.21;  C=0.74 |

Questionnaires: Hospital Anxiety and Depression Scale (HADS), IES= Impact of Events Scale; RPQ= Rivermead Postconcussion Symptoms Questionnaire; * interquartile range odds ratios that correspond to change from 75^th^ to 25^th^ percentile.

**Table 1b. Other recent studies in large cohorts**

| **First author, year** | **Study, country** | **Population, sample size** | **Early symptoms,**  **Measurement**  **, Timing** | **Odds ratios (OR), regression coefficients, standardized mean difference (SMD, “Cohen’s d”) with**  **95% Confidence intervals (CI)** | **Other variables considered** | **Outcome** |
| --- | --- | --- | --- | --- | --- | --- |
| Temkin, ^123^ 2022 | TRACK-TBI, USA | >16 years  Level 1 trauma centres,  majority GCS 13-15,  N= 1718 | PCS (RPQ),  Post-traumatic stress (PCL-5);  2 weeks | RPQ total score >=16: SMD 1.34, 95% CI 1.23 to 1.46  PCL-5 total score >=33: SMD 1.13, 95% 1.00 to 1.26. | Separately sociodemographic and injury-related characteristics and biomarkers: smaller SMDs | 3-month PPCS  (RPQ) |
| Campbell- Sills, ^138^  2021 | TRACK-TBI, USA | GCS 13-15, >16 years,  Level 1 trauma centers,  N=727-883 | PCS (RPQ);  2 weeks | OR* 1.68, 95% CI 1.22 to 2.32 | Age, sex, race/ethnicity, education, cause of injury, hospital admission, psychiatric history, prior TBI, substance abuse | 3-month  suicidal ideation |
| Zahniser, ^139^  2019 | TRACK-TBI and TED, USA | GCS 13-15  N= 717 | Depression (Brief Symptom Inventory, BSI);  2 weeks | λs =-0.21, p<.001 in a cross-legged panel analysis | Age, sex, computed tomography findings, and psychiatric history | 3-month  GOSE |
|  |  |  | Anxiety (BSI);  2 weeks | λs =-0.25, p<.001 in a cross-legged panel analysis | Age, sex, computed tomography findings, and psychiatric history | 3-month  GOSE |

*Standardized odds ratios.

**References to Supplementary Tables:**

**Table 1a**

Stulemeijer M, Van Der Werf S, Borm GF, et al. Early prediction of favourable recovery 6 months after mild traumatic brain injury. J Neurol Neurosurg Psychiatry 2008;79(8):936–942; doi: 10.1136/jnnp.2007.131250.

Van Der Naalt J, Timmerman ME, De Koning ME, et al. Early predictors of outcome after mild traumatic brain injury (UPFRONT): an observational cohort study. Lancet Neurol 2017;16(7):532–540; doi: 10.1016/S1474-4422(17)30117-5.

Cnossen MC, Van Der Naalt J, Spikman JM, et al. Prediction of Persistent Post-Concussion Symptoms after Mild Traumatic Brain Injury. J Neurotrauma 2018;35(22):2691–2698; doi: 10.1089/neu.2017.5486.

Le Sage N, Chauny J-M, Berthelot S, et al. Post-Concussion Symptoms Rule: Derivation and Validation of a Clinical Decision Rule for Early Prediction of Persistent Symptoms after a Mild Traumatic Brain Injury. J Neurotrauma 2022;39(19–20):1349–1362; doi: 10.1089/neu.2022.0026.

Mikolić A, Steyerberg EW, Polinder S, et al. Prognostic Models for Global Functional Outcome and Post-Concussion Symptoms Following Mild Traumatic Brain Injury: A Collaborative European NeuroTrauma Effectiveness Research in Traumatic Brain Injury (CENTER-TBI) Study. J Neurotrauma 2023;40(15–16):1651–1670; doi: 10.1089/neu.2022.0320.

**Table 1b**

Temkin N, Machamer J, Dikmen S, et al. Risk Factors for High Symptom Burden Three Months after Traumatic Brain Injury and Implications for Clinical Trial Design: A Transforming Research and Clinical Knowledge in Traumatic Brain Injury Study. J Neurotrauma 2022;39(21–22):1524–1532; doi: 10.1089/neu.2022.0113.

Campbell-Sills L, Jain S, Sun X, et al. Risk Factors for Suicidal Ideation Following Mild Traumatic Brain Injury: A TRACK-TBI Study. J Head Trauma Rehabil 2021;36(1):E30–E39; doi: 10.1097/HTR.0000000000000602.

Zahniser E, Nelson LD, Dikmen SS, et al. The Temporal Relationship of Mental Health Problems and Functional Limitations following mTBI: A TRACK-TBI and TED Study. J Neurotrauma 2019;36(11):1786–1793; doi: 10.1089/neu.2018.6172.
